# Supplementary material for: Expression of Retroelements in Cervical Cancer and Their Interplay with HPV Infection and Host Gene Expression
Source: Cancers (Basel). 2021 Jul 14;13(14):3513. doi: 10.3390/cancers13143513 (PMC8306386; doi:10.3390/cancers13143513)
Supplement: Supplementary file 1 [file cancers-13-03513-s001.zip › Supplementary figures.pdf]

cancers

MDPI

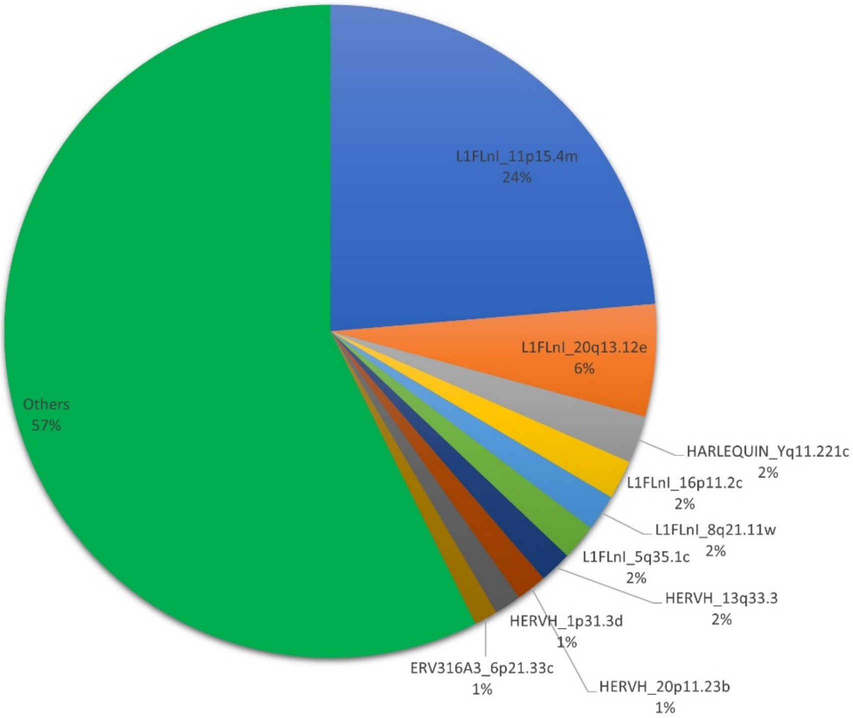

Supplementary Figure S1. Percentage of the most frequent HERV and L1 expressed in cervical cancer samples.

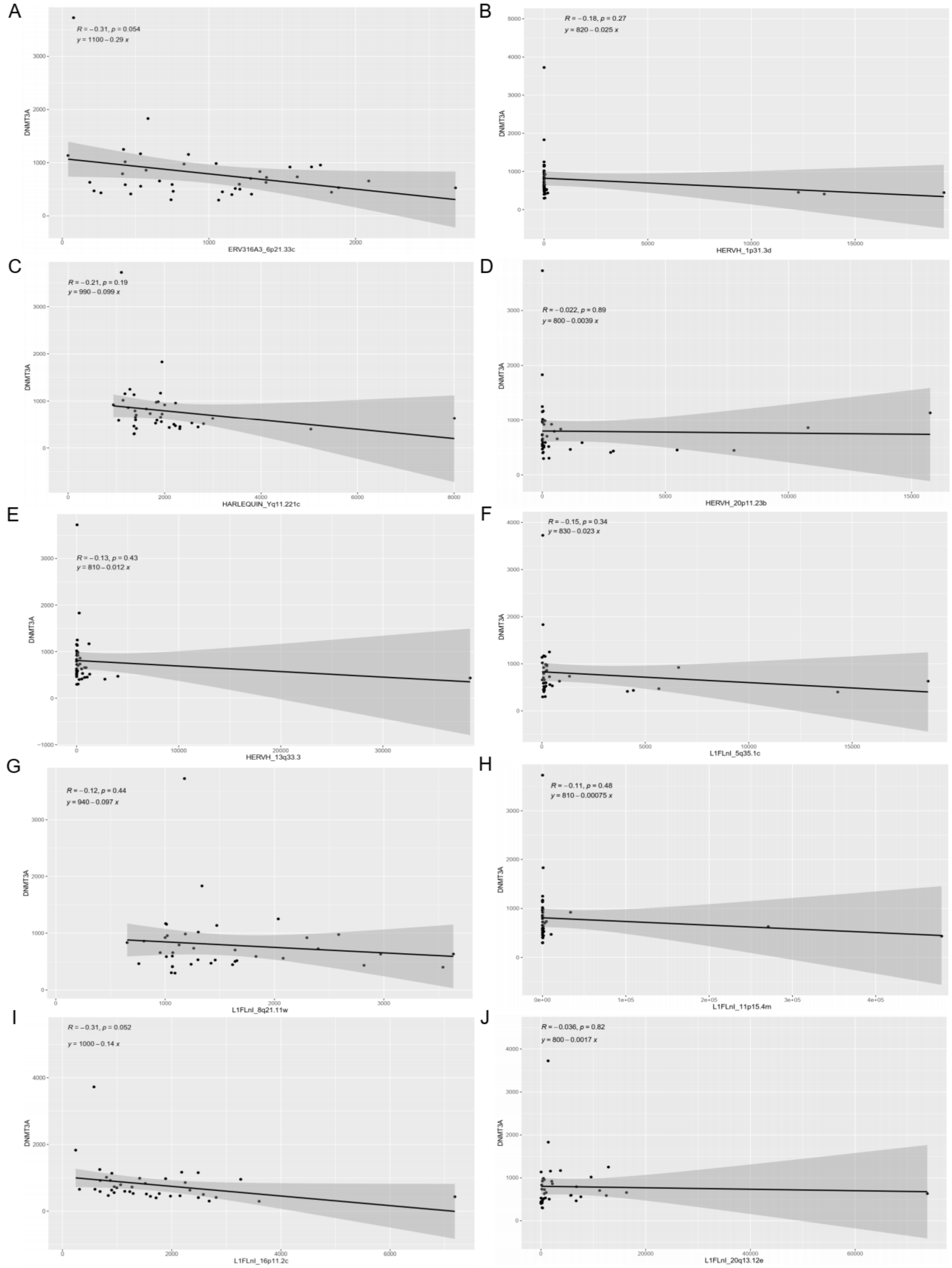

Supplementary Figure S2. Correlation between expression of DNA methyltransferase 3A (*DNMT3A*) and retroelements (A) *ERV316A3_6p21.33c*, (B) *HERVH_1p31.3d*, (C) *HARLEQUIN_Yq11.221c*, (D)

HERVH_20p11.23b, (E) *HERVH_13q33.3*, (F) *L1FLnI_5q35.1c*, (G) *L1FLnI_8q21.11w*, (H) *L1FLnI_11p15.4m*, (I) *L1FLnI_16p11.2c* and (J) *L1FLnI_20q13.12e*. Pearson correlation coefficient (R), p-value (p) and linear equation are shown for each correlation.

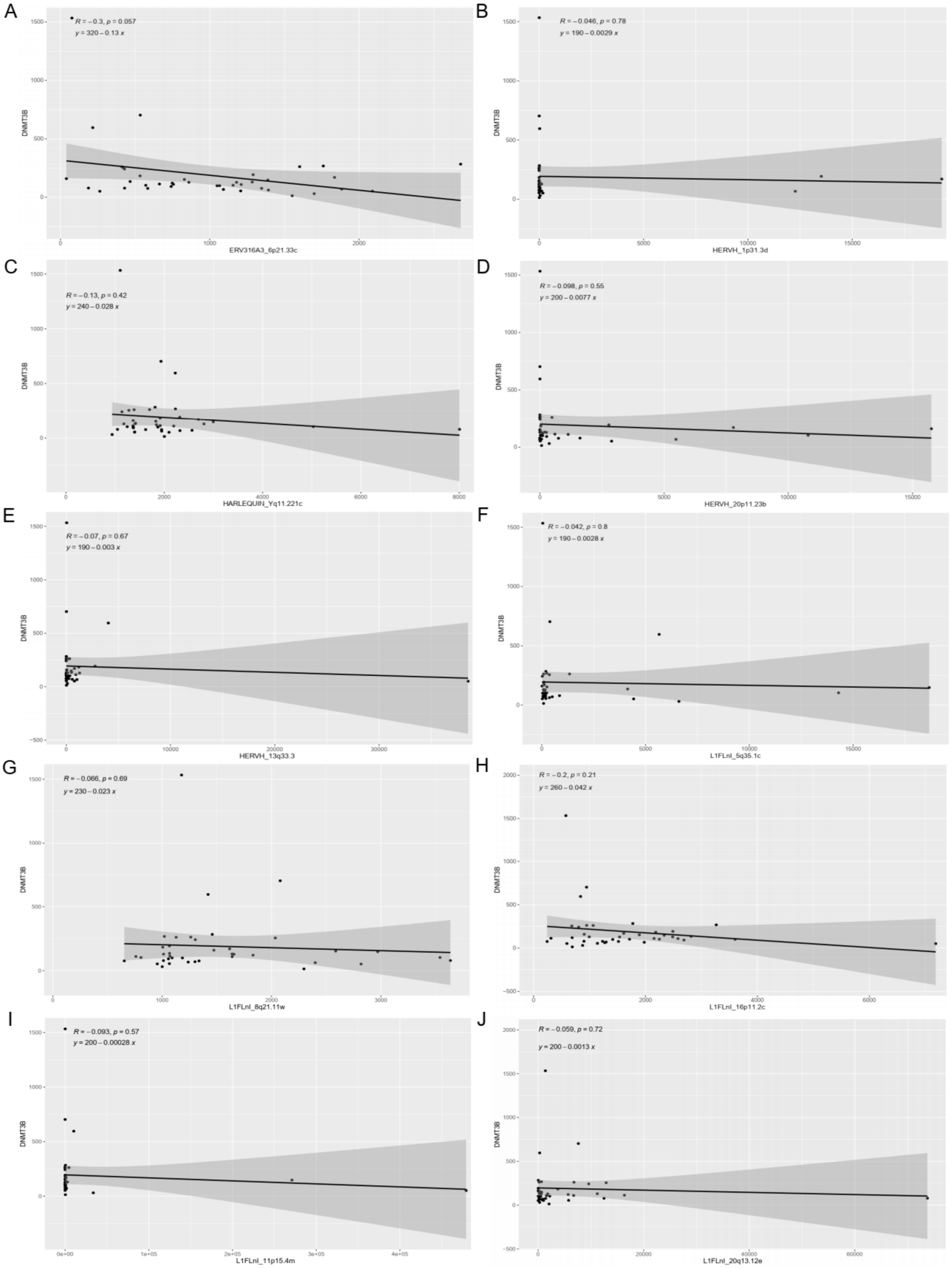

Supplementary Figure S3. Correlation between expression of DNA methyltransferase 3B (*DNMT3B*) and retroelements (A) *ERV316A3_6p21.33c*, (B) *HERVH_1p31.3d*, (C) *HARLEQUIN_Yq11.221c*, (D)

HERVH_20p11.23b, (E) *HERVH_13q33.3*, (F) *L1FLnI_5q35.1c*, (G) *L1FLnI_8q21.11w*, (H) *L1FLnI_11p15.4m*, (I) *L1FLnI_16p11.2c* and (J) *L1FLnI_20q13.12e*. Pearson correlation coefficient (R), p-value (p) and linear equation are shown for each correlation.

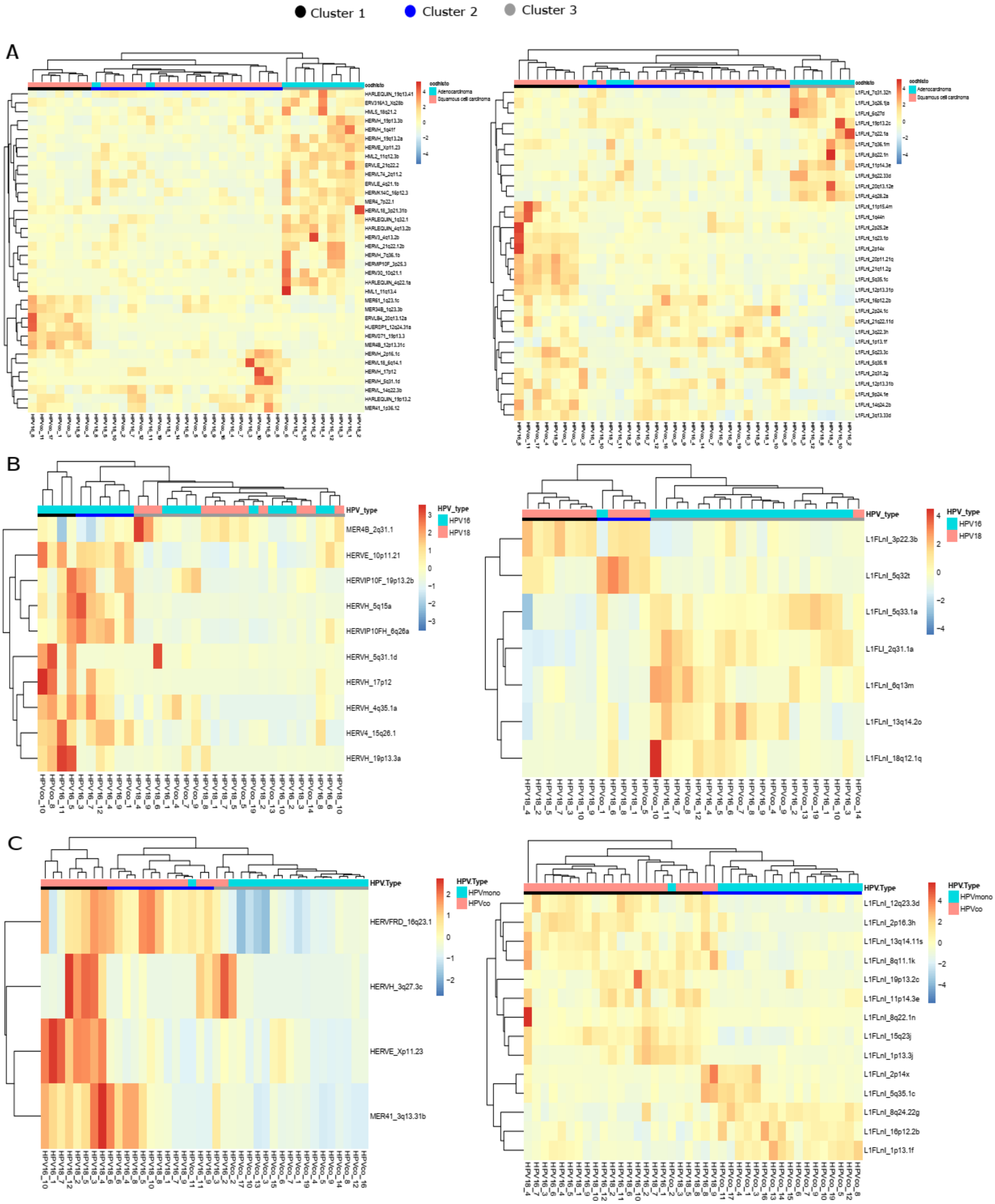

Supplementary Figure S4. HERV (left panels) and L1 (right panels) differentially expressed in cervical cancer. Hierarchical clustering heatmap to HERV and L1 was performed to (A) tumor

histological type, (B) infecting HPV type and (C) HPV co- or monoinfection analysis. Sample clusters are highlighted in black (cluster 1), blue (cluster 2) and gray (cluster 3).
