# Supplementary material for: Expression of Retroelements in Cervical Cancer and Their Interplay with HPV Infection and Host Gene Expression
Source: Cancers (Basel). 2021 Jul 14;13(14):3513. doi: 10.3390/cancers13143513 (PMC8306386; doi:10.3390/cancers13143513)
Supplement: Supplementary file 1 [file cancers-13-03513-s001.zip › Supplementary Table 1_062921.pdf]

Supplementary Table S1 - Differentially expressed HERV genes in cervical cancer according to tumor type and HPV infection

Analysis	HERV family	HERV	log2FoldChange	q-value
Sq x Adeno	ERV1	HERV3_4q13.2b	-475.03	0.00384871260190943
	ERV3	ERV316A3_Xq28b	-236.92	0.00613809572264938
	ERVL	ERVLE_21q22.2	-351.74	0.00730684007155111
		ERVLB4_20q13.12a	397.88	0.00730684007155111
		ERVLE_4q21.1b	-257.82	0.00918947395351183
	HARLEQUIN	HARLEQUIN_19p13.2	483.33	0.000806173152614777
		HARLEQUIN_19q13.41	-176.39	0.00230879701997089
		HARLEQUIN_1q32.1	-314.17	6.60E+09
		HARLEQUIN_4q13.2b	-465.80	0.00283405322433557
		HARLEQUIN_4q22.1a	-551.94	0.00156616104180129
	HERVE	HERV30_10q21.1	-456.93	0.000277606486728701
		HERVE_Xp11.23	-407.20	3.32E+08
	HERVH	HERVH_17p12	6.051	0.00193839791714715
		HERVH_19p13.3b	-210.28	0.00950953053061449
		HERVH_19q13.2a	-480.31	1.60E+08
		HERVH_1q41f	-50.52	0.00104185338555888
		HERVH_2p16.1c	416.96	0.00613809572264938
		HERVH_5q31.1d	789.44	0.000552726123663189
		HERVH_7q36.1b	-547.03	0.00264419009959699
		MER4_7p22.1	-342.91	0.000100232758819264
	HERVI	HERVIP10F_3p25.3	-318.59	0.00156616104180129
	HERVK	HML1_11q13.4	-402.74	0.000806173152614777
		HML2_11q12.3b	-254.35	0.00279956665944161
		HML5_18q21.2	-350.60	0.00568106904059964
		HERVK14C_16p12.3	-273.13	0.00613809572264938
	HERVL	HERVL_14q22.3b	300.10	0.0053176309399731
		HERVL_21q22.12b	-466.19	0.00215253033861125
		HERVL18_3p21.31b	-365.75	0.000976227367374748
		HERVL18_6q14.1	476.40	0.00183126231587784
		HERVL74_2q11.2	-440.67	1.60E+08
	HERVS	HERVS71_19p13.3	373.10	0.0013657120468356
	HUERS	HUERSP1_12q24.31a	512.50	0.00186604485645976
	MER4	MER41_1p36.12	114.27	0.00215253033861125
		MER61_1q23.1c	305.40	0.00317774170948708
		MER34B_1q23.3b	269.61	0.00230879701997089
		MER4B_12p13.31c	34.49	0.00193919224696548
HPV 18 x HPV 16	ERV1	HERV4_15q26.1	-340.147	0.00483732693535448
	HERVE	HERVE_10p11.21	-30.84	0.00147522939780509
	HERVH	HERVH_17p12	-709.38	0.00058324436065384
		HERVH_19p13.3a	-622.93	0.000993525367583365
		HERVH_4q35.1a	-57.97	1.91E+09

		HERVH_5q15a	-710.71	0.00483732693535448
		HERVH_5q31.1d	-770.85	0.000993525367583365
	HERVI	HERVIP10FH_6q26a	-496.98	0.000108226419704014
		HERVIP10F_19p13.2b	-451.60	0.00483732693535448
	MER4	MER4B_2q31.1	137.21	0.00483732693535448
**HPVco x HPVmono**	HERVE	HERVE_Xp11.23	398.38	3.70E+08
	HERVF	HERVFRD_16q23.1	162.86	8.64E+06
	HERVH	HERVH_3q27.3c	819.92	9.71E+07
	MER4	MER41_3q13.31b	37.32	7.68E+07
