# Supplementary material for: Expression of Retroelements in Cervical Cancer and Their Interplay with HPV Infection and Host Gene Expression
Source: Cancers (Basel). 2021 Jul 14;13(14):3513. doi: 10.3390/cancers13143513 (PMC8306386; doi:10.3390/cancers13143513)
Supplement: Supplementary file 1 [file cancers-13-03513-s001.zip › Supplementary table 2.pdf]

Supplementary Table S2 - Retroelements nearby HPV integration sites

Sample	HPV type	localization	HPV orientation	HERV loci	HERV orientation	HERV distance to HPV	L1 loci	L1 orientation	L1 distance to HPV
HPVco_7	HPV16	11p15.1	antisense	HERVH_11p15.1a; HUERSP3_11p15.1	sense; sense	1.307kb; 20 kb	x	x	x
HPV16_5	HPV16	13q22.1	antisense	x	x	x	L1FLnL_13q22.1a; L1FLnL_13q22.1b	antisense; sense	837 kb; 549 kb
HPV18_6	HPV18	20q13.13	antisense	MER4_20q13.13; MER101_20q13.13	sense; sense	333 kb; 67kb	x	x	x
HPVco_8	HPV16	22p13.2	antisense	ERVLE_22q13.2f; ERVLE_22q13.31a	sense; antisense	113 kb; 2.118 kb	x	x	x
