# Supplementary material for: Expression of Retroelements in Cervical Cancer and Their Interplay with HPV Infection and Host Gene Expression
Source: Cancers (Basel). 2021 Jul 14;13(14):3513. doi: 10.3390/cancers13143513 (PMC8306386; doi:10.3390/cancers13143513)
Supplement: Supplementary file 1 [file cancers-13-03513-s001.zip › Supplementary Table 3 .pdf]

Supplementary Table S3 - Interleukin-20 gene family nearby retroelements expressed in cervical cancer

GENE	HERV	L1
IL19	ERVLE_1q32.1e	L1FLnI_1q32.1n
IL20	x	L1FLnI_1q32.1n
IL20RA	HERVH_6q23.3	x
IL20RB	x	L1FLnI_3q22.3i
	x	L1FLnI_3q22.3k
	x	L1FLnI_3q22.3l
	x	L1FLnI_3q22.3h
	x	L1FLnI_3q22.3g
IL22RA2	HERVH_6q23.3	x
